# Well-Being and Perceived Quality of Life in Elderly People Displaced After the Earthquake in L’Aquila, Italy

**DOI:** 10.1007/s10900-013-9793-7

**Published:** 2013-12-04

**Authors:** Anna Rita Giuliani, Antonella Mattei, Flavio Santilli, Giovanna Clori, Maria Scatigna, Leila Fabiani

**Affiliations:** Department of Medicine, Health and Environment Sciences, University of L’Aquila, Viale S. Salvatore Edificio 6, 67100 L’Aquila, Italy

**Keywords:** Earthquake, Elderly, Quality of life, Social relationships

## Abstract

On 6 April 2009, the city of L’Aquila was hit by a violent earthquake that destroyed almost all of its medieval centre, and the surviving inhabitants were evacuated and relocated in temporary quarters or undamaged homes. The aim of this study was to investigate the perceived quality of life of the elderly population 3 years after the earthquake in relation to the social and logistic issues of new housing. The study was carried out between October 2011 and March 2012, and involved 571 subjects aged over 65 years living in the municipality of L’Aquila. The interviews took place in the surgeries of general practitioners and the city’s Department of Prevention and Vaccination in the anti-influenza immunisation period. The instrument used was a 36-item questionnaire with closed, multiple choice answers divided into the following sections: demographics, everyday activities, health and perceived health, and the quality of life in the city. The results show that, 3 years after the earthquake, the elderly population living in the new towns and temporary housing of L’Aquila have a worse perception of their quality of life than the others. They feel a certain social isolation and wish to live elsewhere. Governments faced with the problems arising from a natural calamity should take into account all of the elements making up a good quality of life and, before making choices whose impact cannot be changed, consider both their immediate and long-term social consequences.

## Introduction

A destructive earthquake gives rise to a collective sense of mourning due to the loss of affective relations, material goods and everyday points of reference, and a consequent need to redefine living patterns. The disruption of people’s lives and their loss of certainties affect their physical and mental health in both the short term and years after the calamity [1–3]. The literature is full of papers describing the presence of post-traumatic stress disorder (PTSD) [4–16] and depressive disturbances (major depression MD, other), anxiety, irritability and insomnia [3, 7, 11, 14, 16–29] and there is also an increase in the incidence chronic degenerative diseases and a worsening in pre-existing conditions [2, 10, 17, 25]. All of these factors have a negative impact on the quality of life [2, 15, 30], which is also affected by gender, age, physical injury, and damaged housing with subsequent displacement and the dissolution of social networks [10, 11, 13, 20, 24, 25, 31–33].

The elderly are among the most affected [10, 11, 13, 17, 21, 24, 26, 27, 29, 34, 35] because, as pointed out by the WHO in its report “*Active aging: a policy framework*” (2002), their quality of life not only depends on their state of health, but also on many other variables such as their social relations, their recreational and cultural activities, and the environment. These can all play a protective role in active aging, but their absence is a risk factor for physical and cognitive decline.

It is for this reason that we decided to investigate the elderly living in the highly urbanised area of L’Aquila which, on 6 April 2009, was struck by an earthquake (magnitude 6.3 on the Richter scale) that almost totally destroyed the city’s medieval centre and its immediate surroundings. After the emergency phase, some of the people whose homes had been destroyed were relocated to 19 areas with earthquake-resistant housing, which led to their decentralisation and the disruption of their social networks. They found themselves living in unfamiliar surroundings devoid of services that are still little more than dormitories almost 4 years after the earthquake. This has had a profound effect on the population as a whole, but particularly on the elderly, who are less self-sufficient in terms of mobility and, having been deprived of friendships, social gatherings and places of worship, are now isolated and unable to re-establish their everyday lives or their contacts with friends and relations. The aim of the study was to verify the impact of the change on their perceived quality of life.

## Materials and Methods

The study was carried out between October 2011 and March 2012, and involved 571 subjects aged over 65 years living in the municipality of L’Aquila. It investigated various factors that are important for the quality of life of the elderly (health, family relationships, peacefulness and the level of satisfaction with life) and the effect that the earthquake of 2009 has had on these variables. The interviews, which were conducted by post-graduate physicians specialising in Hygiene and Preventive Medicine, took place in the surgeries of general practitioners (GPs) and the city’s Department of Prevention and Vaccination in the anti-influenza immunisation period. The GPs invited the over-65 s in their surgeries to undergo the interviews, and all of those who agreed were consecutively enrolled. The interviews were conducted anonymously and all of the interviewees were asked to give their written informed consent after the purpose of the study had been explained to them.

The standardised instrument was based on one published and validated questionnaire used within a multicentric Italian study carried on by National Research Council (CNR) [36], and consisted of 36 questions with closed, multiple choice answers, divided into the following sections: demographics, everyday activities, health, perceived well-being [using two visual analogue scales (VAS): happiness/well-being and physical well-being], and the quality of life in the city.

Before being administered to the study sample, the questionnaire was submitted to a small group of elderly for testing-retesting, the result of which was a Cohen’s *kappa* of 0.91.

### Statistical Analysis

The characteristics of the study sample were analysed using descriptive statistics. The discrete and nominal variables were expressed in terms of frequency and percentages, and the differences were tested using the χ^2^ or Fisher’s exact test. The continuous variables were expressed as mean values and standard deviations. The normality of data distribution was tested using the Shapiro–Wilk test. Wilcoxon–Mann–Whitney test was used to evaluate differences by gender and housing conditions on the basis of the VAS score (1–10) for each individual’s perceived quality of life. Multivariate logistic regression models were used to estimate the odds ratios (ORs) and 95 % confidence intervals of the associations between the need of a subject to leave his/her current housing conditions (outcome variable) and the relative contributions made by some demographic, living and perceived well-being variables (the explanatory variables). The propensity to socialise or not was analysed on the basis of the VAS scores of the interviewees ≥5 means the presence of perceived happiness/well-being and ≤4 its absence. The tests were two-sided and a *p* value of 5 % was considered significant. Spearman’s coefficient was used to measure the degree of correlation between the answers to two questions, “How is your health in general?” expressed with a score between 1 (very bad) to 5 (Very Good) and “Using a scale from 1 to 10, could you tell me how happy/content you feel in this period of your life? “.The data were analysed using the STATA/IC 12.1 statistical package.

The study protocol has been submitted to the institutional review board for ethical issues approval.

## Results

Sociodemographic characteristics of the sample of 571 elderly, which included more men than women (50.44 vs 49.56 %) and had a mean age of 73 years (most of the subjects were aged 65–74 years are shown in Table 1. About 70 % were living with their spouses, and there were more widows than widowers (68.38 %). Their educational level was medium–low: the women were generally less educated, but there were more of them at the two extremes. The vast majority (97.70 %) were living on a pension of one kind or another, and only 1.06 % declared that they were working. Almost a third (31.87 %) were living in temporary homes provided by the government.

**Table 1:** Sociodemographic characteristics of the sample by gender (n = 571)

	Total 571	Males	Females	*p* value*
		n (%) 288 (50.44)	n (%) 283 (49.56)	
Age (years)	570	288	282	n.s
65–74	325 (57.02)	160 (49.23)	165 (50.77)	
75–79	141 (24.74)	76 (53.90)	65 (46.10)	
80–84	83 (14.56)	40 (48.19)	43 (51.81)	
>84	21 (3.68)	12 (57.14)	9 (42.86)	
Marital status	570	288	282	0.000
Single	22 (3.86)	12 (54.55)	10 (45.45)	
Married	402 (70.53)	228 (56.72)	174 (43.28)	
Widowed	136 (23.86)	43 (31.62)	93 (68.38)	
Separated	7 (1.23)	2 (28.57)	5 (71.43)	
Divorced	3 (0.53)	3 (100)	0 (0.00)	
Education	567	285	282	0.006
Primary school	187 (32.98)	76 (40.64)	111 (58.36)	
Middle school	212 (37.39)	114 (53.77)	98 (46.23)	
High school	141 (24.87)	83 (58.87)	58 (41.13)	
University	27 (4.76)	12 (44.44)	15 (55.56)	
Source of income	566	287	279	0.000
Salary	6 (1.06)	4 (66.67)	2 (33.33)	
Employment pension	495 (87.46)	271 (54.75)	224 (45.25)	
Employment + disability pension	14 (2.47)	8 (57.14)	6 (42.86)	
Disability pension	14 (2.47)	4 (28.57)	10 (71.43)	
Economic support	7 (1.24)	0 (0.00)	7 (100)	
Widow/dependents’ pension	30 (5.30)	0 (0.00)	30 (100)	
Current housing	571	288	283	n.s
Rented/owned housing	389 (68.13)	197 (50.64)	192 (49.36)	
Temporary housing	182 (31.87)	91 (50.00)	91 (50.00)	

* χ^2^ or Fisher’s exact test

*n.s* not statistically significant (*p* > 0.05)

Table 2 shows their perceived mean level of happiness/well-being before and after the earthquake, as measured by a 1–10 VAS. The scores recorded by the subjects living in temporary housing were lower than those of the subjects living in rented or owned accommodation (5.43 vs 5.82; *p* = 0.013). The scores attributed to the pre-earthquake period were higher for both groups but particularly the former (7.85 vs 7.61), although the difference was not statistically significant. When asked to judge the happiness of people of the same age, the subjects living in temporary housing gave lower scores than those living in rented/owned housing (5.14 vs 5.35; *p* = 0.01), and lower than those attributed to themselves. Analysis of this perception by gender showed that the women had a more negative view of their well-being than the men both before (7.56 vs 7.82; *p* = 0.0015) and after the earthquake (5.40 vs 5.98; *p* = 0.000). Although not statistically significant, the subjects’ perception of the happiness/well-being of people of the same age maintained the male/female difference, and the scores were once again lower than those attributed to themselves.

**Table 2:** Perception of happiness/well-being by type of housing and gender

	Sample as a whole			Rented/owned housing			Temporary housing			*p* value*
	Total	Mean	SD	No.	Mean	SD	No.	Mean	SD	
Using a score from 1 to 10, could you tell me how happy/content you feel in this period of your life?	571	5.69	1.61	389	5.82	1.64	183	5.43	1.52	0.013
Using a score from 1 to 10, how happy do you think people of your age are?	513	5.29	1.10	352	5.35	1.11	161	5.14	1.08	0.01
Again using a score from 1 to 10, could you tell me how happy/content you felt before 6 April 2009?	571	7.69	1.61	388	7.61	1.53	183	7.85	1.10	n.s.

	Sample as a whole			Males			Females			*p* value*
	Total	Mean	SD	No.	Mean	SD	No.	Mean	SD	
Using a score from 1 to 10, could you tell me how happy/content you feel in this period of your life?	571	5.69	1.61	288	5.98	1.61	283	5.40	1.56	0.000
Using a score from 1 to 10, , how happy do you think people of your age are?	513	5.29	1.10	256	5.38	1.13	256	5.20	1.07	n.s.
Again using a score from 1 to 10, could you tell me how happy/content you felt before 6 April 2009?	571	7.69	1.61	287	7.82	1.49	283	7.56	1.30	0.0015

* *p* < 0.05: statistically significant differences by housing status and gender (Wilcoxon–Mann–Whitney test)

*n.s.* not statistically significant (*p* > 0.05)

The interviewees were then asked whether they would like to live in another area or city, and 25.09 % (n = 142) said they would. Putting this wish in relation to the subjects’ perceived well-being and housing conditions (Table 3), our data show that the probability of wishing to live in a different place was higher as the score attributed to the subjects’ perception of their current happiness/well-being diminished (OR 0.77; 95 % CI 0.65–0.92), and the score attributed to their well-being before the earthquake increased (OR 1.39; 95 % CI 1.15–1.69). The association between the wish to live elsewhere and the score attributed to the happiness of people of the same age was not significant. The probability of a wish to move was very high among the subjects living in temporary housing (OR 8.23; 95 % CI 5.39–12.55).

**Table 3:** Associations between the 142 people who responded positively to the question “Would you like to live in a different area/city?”, their perceived well-being and their housing conditions

	OR	95% CI
Using a score from 1 to 10, could you tell me how happy/content you feel in this period of your life?	0.77*	0.65–0.92
Using a score from 1 to 10, how happy do you think people of your age are?	1.06	0.85–1.33
Again using a score from 1 to 10, could you tell me how happy/content you felt before 6 April 2009?	1.39*	1.15–1.69
People living in temporary housing	8.23*	5.39–12.55

* Statistically significant association

We then asked: “Where would you like to live?” The answer “In my previous area” was chosen by 58.61 % of the sample, whereas 2.87 % would change it, 10.66 % would move to another city, and 28.87 % had no preference. In answer to the question “With whom do you spend your free time?”, 25.09 % said with friends (mainly men: 68.53 vs 31.47 %), and only 4.56 % with their neighbours. Relationships with neighbours were declared to be formal (51.49 %), reciprocally supportive (2.11 %), conflictual (1.05 %) and good (45.34 %) (data not shown).

Table 4 shows the association between happiness/well-being and the propensity to engage in more or less socialising activities. It can be seen that those with a greater perception of their own well-being (a VAS score of ≥5) were more likely to engage in both socialising (sport/recreational activities, visits to relatives and friends) and non-socialising activities (reading books, manual and agricultural work) than those with a score of ≤4.

**Table 4:** Propensity to engage in more or less socialising activities during free time in relation to the scores attributed to happiness/well-being

	Poor perception of happiness/well-being	Good perception of happiness/well-being	*p* value*
I watch television	203 (90.63)	317 (92.15)	n.s.
I read a book	89 (39.38)	189 (54.62)	0.000
Agricultural work	53 (23.45)	118 (34.10)	0.007
Manual work	40 (17.70)	80 (23.12)	n.s.
Walking/shopping	124 (54.87)	227 (65.61)	0.010
Practice sport	15 (6.64)	54 (15.61)	0.001
Recreational activities	11 (4.87)	33 (9.54)	0.040
Visits to relatives	134 (59.29)	245 (70.81)	0.004
Visits to friends	46 (20.35)	111 (32.08)	0.002
I go to the park	47 (20.80)	71 (20.52)	n.s.
Religious activities	98 (43.56)	171 (49.52)	n.s.

* *p* < 0.05: statistically significant difference using χ^2^ or Fisher’s exact test

*n.s.* not statistically significant

The analysis of the correlation between the perception of well-being and health status shows that an increase of the scores given to their happy/content also improves the judgment of his state of health (r = 0.45, *p* = 0.000) (data not showed).

## Discussion

The aim of our survey of the perceived quality of life of a sample of elderly people (mean age 73 years) was to evaluate their state of well-being 3 years after the earthquake. The sample corresponded to about 4 % of the over-65 year olds living in the municipality.

The analysis was based on the VAS scores of happiness/well-being attributed to themselves by the subjects as a measure of their perceived quality of life, and this measure was used to evaluate the possible variables determining their health and social welfare (that also in our samplecorrelate), particularly social activities and housing conditions as changing residence was the principal intervention after the earthquake. The relationship between the trauma and morbidity seems to be clear, and the effect of the medium-term change in housing conditions on the perception of well-being is equally important.

The earthquake that struck L’Aquila in April 2009 has had considerable repercussions on the health of the people affected. Numerous studies carried out after the earthquake have revealed a 37 % increase in the prescriptions of anti-depressants and a 129 % increase in the prescriptions of antipsychotics [37], particularly among elderly women. Among the young, there has been an increase in the consumption of alcohol, tobacco and cannabis [38]. A study of a sample of subjects aged 18–69 years carried out by the Istituto Superiore di Sanità in 2010 [16] found that, in comparison with 2007–2008, the occurrence of PTSD had increased ten times, that of depression had increased six times, and that of sedentariness (39 %) had doubled, mainly among over-50 year olds (47 %), women (42 %) and relocated (42 %). Economic and employment difficulties are the common denominator of both PTSD (4.1 %) and MD (5.8 %) [14]. Furthermore, immediately after the earthquake, there was a 26.9 % increase in the number of hospitalisations due to cardiovascular diseases among the elderly [39].

Our data indicate that the over-65 s experience a certain social isolation: most of their free time is spent with their families (81.75 %), and their relationships with friends and neighbours are rather formal, as shown by the absence of conflicts and support. It is also necessary to bear in mind that only 1.06 % of our sample worked, which suggests that the intervewees have a lot of free time: however, this is not used for socialising as very few subjects engage in activities such as sport or recreation that would bring them into contact with their contemporaries. Only 25.09 % declared they spend their free time with friends, and no more than 4.56 % with their neighbours, a finding that is in line with the results of the 2010 survey reporting anhedonia and depression in 10 % of the interviewees [16].

The isolation of the elderly population may be due to their relocation destroying their social networks. This view is supported by the findings of other studies of people involved in natural calamities, which have shown that changes in housing conditions, altered social networks, and living far from friends and relatives increase psychiatric and quality of life disturbances [1, 7, 11, 12, 15, 20, 26, 31, 33] whereas social support improves both [13, 18, 21, 22, 30]. Housing conditions also affected our subjects’ perception of their happiness/well-being which, logically enough, worsened after the earthquake particularly among those living in temporary housing (Table 2). Analysing the data by gender showed that the women had a more negative perception of life both before (*p* < 0.0015) and after the earthquake (*p* < 0.000) (Table 2). This is in line with the findings of other studies showing that women are more affected by the consequences of an earthquake [5, 8, 14, 20, 22–24, 26, 30], as well as those of a recent study showing that psychiatric morbidity is greater among relocated women than women who have not had to move [40]. The more negative the perception of their well-being, the greater the wish of our subjects to live elsewhere. This was particularly true of the subjects living in the new towns (Table 3), who longed to be able to return to their earthquake-damaged homes.

It is worth noting that, when the interviewees were asked to judge the happiness/well-being of their contemporaries, the scores were lower than those attributed to themselves, even when the variables of housing conditions and gender were taken into account. As pointed out in other studies [1, 3, 12, 26, 30, 32], this is unlikely to be due to economic factors because almost all of our interviewees were living on a pension, and this would not have been changed by the earthquake.

We can therefore state that the nexus between the change in housing conditions and the quality of life and social activities is sufficiently well documented. The creation of new residential areas in L’Aquila certainly responded to immediate need for housing, but this was not followed up by the subsequent phase of creating the services and places of socialisation that could have restored a minimum of social fabric. There has been a lack of the “weak ties” that would have allowed the elderly to confront the difficulties caused by the earthquake and their consequent relocation, and an absence of sharing the problems created by the new situation. In the case of a calamity, public assistance should take into account the particular characteristics of different age groups. As they have ceased their active role in society, the everyday life of the elderly is based on affective and relational factors, which help them to overcome their difficulties and allow them to feel themselves an integral part of the community. After the first period, during which the people of L’Aquila received incredible support from voluntary workers, the second period of relocation interrupted newly formed relationships without restoring the old, also because it was not possible for families/social groups to coordinate their destinations. L’Aquila still has 30,000 inhabitants without a permanent home and it is expected that the reconstruction will take about another 20 years. Many of our interviewees may not be able to return to their homes, and their awareness of this affects their health, mood and desire to socialise. Governments faced with the problems arising from a natural calamity should take into account all of the elements making up a good quality of life and, before making choices whose impact cannot be changed, consider both their immediate and long-term social consequences.
